# Intracellular human antibody fragments recognizing the VP35 protein of Zaire Ebola filovirus inhibit the protein activity

**DOI:** 10.1186/s12896-019-0554-2

**Published:** 2019-09-05

**Authors:** Michela Flego, Aldo Frau, Luisa Accardi, Alessandra Mallano, Alessandro Ascione, Mara Gellini, Elisa Fanunza, Stefano Vella, Paola Di Bonito, Enzo Tramontano

**Affiliations:** 1 Istituto Superiore di Sanità (ISS), National Center for Global Health, Viale Regina Elena 299, 00161 Rome, Italy; 2 Department of Life and Environmental Sciences, University of Cagliari, Cittadella Universitaria di Monserrato SS554 09042 Monserrato, Cagliari, Italy; 3 Department of Infectious Diseases, Viral Hepatitis, Oncoviruses and Retroviruses (EVOR) unit, Istituto Superiore di Sanità (ISS), Viale Regina Elena 299, 00161 Rome, Italy

**Keywords:** Zaire ebolavirus, VP35, scFv, Intrabody

## Abstract

**Background:**

Ebola hemorrhagic fever is caused by the Ebola filovirus (EBOV), which is one of the most aggressive infectious agents known worldwide. The EBOV pathogenesis starts with uncontrolled viral replication and subversion of both the innate and adaptive host immune response. The multifunctional viral VP35 protein is involved in this process by exerting an antagonistic action against the early antiviral alpha/beta interferon (IFN-α/β) response, and represents a suitable target for the development of strategies to control EBOV infection.

Phage display technology permits to select antibodies as single chain Fragment variable (scFv) from an artificial immune system, due to their ability to specifically recognize the antigen of interest. ScFv is ideal for genetic manipulation and to obtain antibody constructs useful for targeting either antigens expressed on cell surface or intracellular antigens if the scFv is expressed as intracellular antibody (intrabody) or delivered into the cells.

**Results:**

Monoclonal antibodies (mAb) in scFv format specific for the EBOV VP35 were isolated from the ETH-2 library of human recombinant antibodies by phage display technology. Five different clones were identified by sequencing, produced in *E.coli* and expressed in CHO mammalian cells to be characterized in vitro. All the selected scFvs were able to react with recombinant VP35 protein in ELISA, one of the scFvs being also able to react in Western Blot assay (WB). In addition, all scFvs were expressed in cell cytoplasm as intrabodies; a luciferase reporter gene inhibition assay performed in A549 cells showed that two of the scFvs can significantly hamper the inhibition of the IFN-β-induced RIG-I signaling cascade mediated by EBOV VP35.

**Conclusion:**

Five antibodies in scFv format recognize an active form of EBOV VP35 in ELISA, while one antibody also recognizes VP35 in WB. Two of these scFvs were also able to interfere with the intracellular activity of VP35 in a cell system in vitro. These findings suggest that such antibodies in scFv format might be employed to develop therapeutic molecules able to hamper EBOV infections.

## Background

Ebola hemorrhagic fever caused by the EBOV is one of the most aggressive zoonoses affecting humans, leading to death within a few days of the exposure [1]. Six species of EBOV are known to date, named after the geographical region in which they were first isolated: Bundibugyo, Reston, Sudan, Taï Forest (formerly Côte d’Ivoire EBOV), Zaire and Bombali EBOV. Sudan, Taï Forest, and Zaire EBOV are responsible for outbreaks in humans, whereas Reston EBOV infects non-human primates, and Bombali virus was recently discovered in bats [2, 3]. Fatal EBOV infections are characterized by rapid viral replication combined with an inadequate antiviral response. Hallmarks of fatal cases are immune suppression with T cells levels below the normal level, no CD8 T cell activation, delay of antibody response in the blood, and high viremia (10^10^ genome copies/ml serum). The EBOV pathogenesis starts with the subversion of both innate and adaptive immune response and the consequent induction of harmful inflammatory responses and tissue necrosis due to disseminated infections [4, 5].

EBOV has a linear, single-stranded, negative RNA genome about 19,000 nucleotides in length. It is composed of seven genes coding for eight proteins in this order: NP (encoding the nucleoprotein), VP35, VP40, GP (encoding the glycoproteins), VP30, VP24, L (encoding the polymerase). The GP gene codes for the two molecular forms GP1 and GP2, generated by RNA editing [1].

VP35 is a conserved multifunctional protein which is a cofactor of the viral RNA polymerase complex along with the NP, VP30, and L protein. Its activity starts at an early stage of the EBOV infection; it is also a double-stranded RNA-binding protein shown to be implicated in hampering the innate immune response [6] by blocking the IFN-mediated antiviral activity through multiple inhibitory effects which include disruption of the RIG-1 pathway by preventing IRF-3 phosphorylation [7, 8], and inhibition of activation of the IFN-inducible dsRNA and Dicer-dependent protein kinase R [9].

Antibody phage display technology makes it possible to select from an artificial immune system human antibodies in scFv format capable of specifically recognizing an antigen of interest. ScFvs, consisting of the VH and VL chain regions of a whole immunoglobulin (Ig), are the smaller portion still retaining the binding properties of the parental Ig. This format is ideal for genetic manipulation in order to obtain antibody constructs potentially useful for diagnostic and therapeutic applications [10]. Furthermore, scFv antibodies can be expressed inside the cell as intrabodies so as to bind to their intracellular target antigen [11]. The two main mechanisms underlying the efficacy of intrabodies are: 1) knockdown of the activity of cytosolic antigens through cytosolic intrabodies [12–14]; 2) diverting of antigens from their natural intracellular compartment by scFv binding due to an extra-signal for intracellular localization [15–17]. Intrabodies can be used to reveal the function of proteins by interfering with their function, although the possibility of targeting intracellular antigens gives them a therapeutic potential for a number of viral infections [18, 19], neurological diseases [20, 21] and cancers [12, 13, 22, 23].

This study reports the selection by phage display and characterization of 5 different human scFv antibodies binding to an active form of the Zaire EBOV VP35 [24, 25]. The ability of these scFvs, expressed as cytosolic intrabodies, to reverse the inhibition of type I IFN induction by intracellular expression of VP35 was tested by a luciferase reporter gene inhibition assay in A549 cells treated with dsRNAs [26].

## Results

### Isolation and characterization of EBOV VP35-specific antibodies

**Fig. 1:**
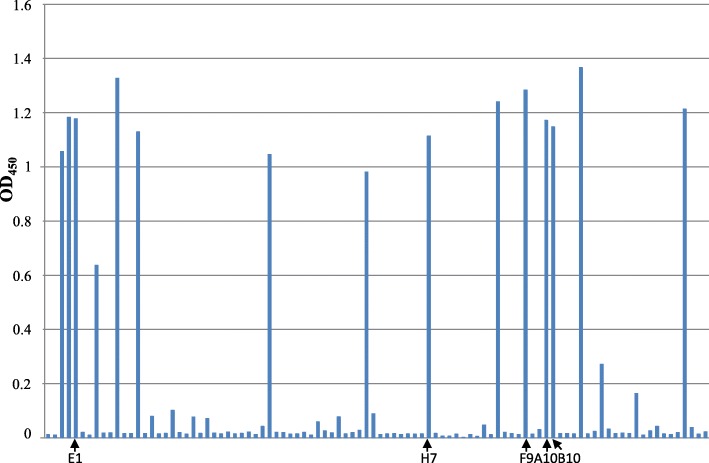
Reactivity of the scFvs against the recombinant EBOV VP35 in ELISA. The IPTG-induced bacterial supernatants of individual colonies from the third round of selection were tested in 96-well microtiter plates coated with the recombinant VP35 protein as an antigen. The cut-off value separating positive from negative samples was calculated as 3 standard deviation values (SD) above the mean, of the values obtained using the irrelevant anti-GO scFv (OD_450_ = 0.079). The five different positive clones isolated, identified by sequence analysis, are indicated

**Fig. 2:**
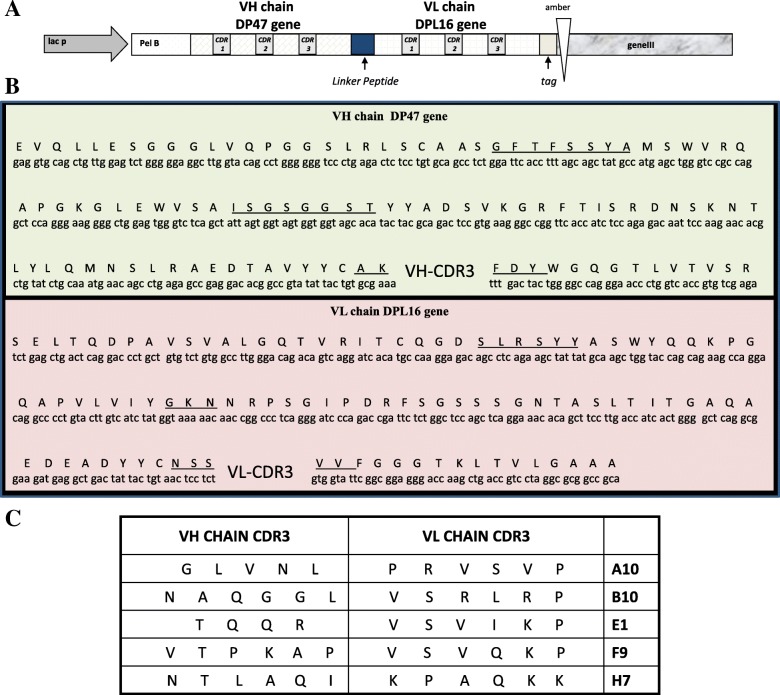
Molecular characterization of the CDR3 belonging to the selected anti-EBOV VP35 scFvs. **a** Schematic representation of the scFv gene in the phage display cassette showing the position of the variable CDR3 in the VH and VL chains. Lac p: Lac promoter; PelB: peptide leader for secretion in the bacterial periplasm. **b** Sequences of the DP47 VH gene and DPL16 VL gene are shown. CDR1, CDR2, CDR3, identified through IMGT (international ImMunoGeneTics information system)/V-Quest analysis are underlined. **c** Amino acid composition of the CDR3 regions in the VH and VL domains of the five most reactive anti-VP35 scFvs

**Fig. 3:**
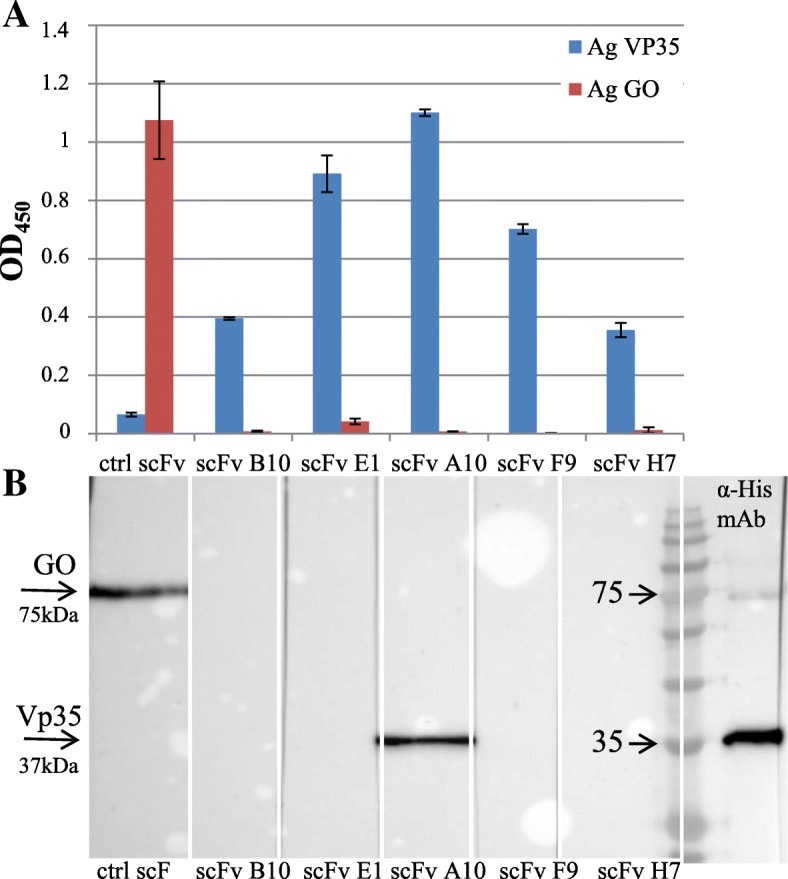
**a** Reactivity of the anti-VP35 scFv clones in ELISA. Either the recombinant VP35 (blue bars) or the recombinant glucose oxidase (GO, red bars) were used as antigens. The anti-GO scFv was used as control (ctrl) for detection in parallel with the anti-VP35 scFvs. The mean values and the SD of a representative experiment out of at least three, performed in triplicate, are reported. **b** Reactivity of the anti-VP35 scFv clones in immunoblot assay. Recombinant VP35 protein and GO antigen as a control were analyzed by SDS-PAGE, blotted onto nitrocellulose membrane and cut into strips. Each strip was incubated with the supernatant deriving from a bacterial clone producing one of the anti-VP35 scFvs as indicated, and the antigen-antibody reaction revealed by ECL. The GO antigen detection by ctrl scFv and the recombinant VP35 detection by anti-His mAb, were used as positive controls. The molecular weights (MW) of the bands corresponding to GO and VP35 protein are indicated by arrows

In order to isolate antibodies specific for the recombinant VP35 expressed in *E.coli* and purified in an active form [24, 27], an approach based on the phage display technology was used. In the ETH-2 library which we used, the diversity (about 10^8^ clones) has been introduced in the complementary-determining region 3 (CDR3) of both the variable heavy chain (VH) and variable light chain (VL) domains [28]. To recover antigen-specific antibody phages, an aliquot of the ETH-2 antibody library containing 10^12^ cfu phage was used for the panning procedure as described elsewhere [29, 30]. In Fig. 1 soluble scFvs derived from IPTG-induced colonies were screened by ELISA to find those specific for the VP35 protein. All the *E. coli* colonies corresponding to the clones exhibiting an OD λ value in ELISA higher than 0.079, were grown and subjected to DNA extraction and sequence analysis. Several clones had identical nucleotide sequences and five different clones, namely B10, A10, E1, F9 and H7, were identified; the amino acid composition of the complete sequences of the VH and VL domains is shown in Fig. 2 along with the schematic representation of a scFv gene in the phagemid cassette. The scFv reactivity towards VP35 was further characterized by ELISA (Fig. 3, panel a) and WB (Fig. 3, panel b) using VP35 recombinant antigen. The protein Glucose Oxidase (GO) and an anti-GO scFv for detection were used as negative controls. The anti-VP35 reactivity in ELISA was confirmed for all B10, A10, E1, F9 and H7 scFvs, while in WB the positivity was only observed for scFv A10, which reacted with a 37 kDa protein identified as the recombinant His-tagged VP35 protein also detected by the anti-His mAb. The scFvs B10, E1, F9, H7 recognized their antigen in ELISA but showed no reactivity in denaturing conditions of WB, suggesting that they probably recognize conformational epitopes. For its part, A10 is still reactive in WB and probably recognizes a linear epitope. The specificity of the anti-VP35 reactivity of B10, A10, E1, F9 and H7 scFvs is confirmed by the observation that they did not react with the irrelevant GO antigen either in ELISA or in WB (Fig. 3).

### Cloning of the scFv genes and their expression in eukaryotic cell system

**Fig. 4:**
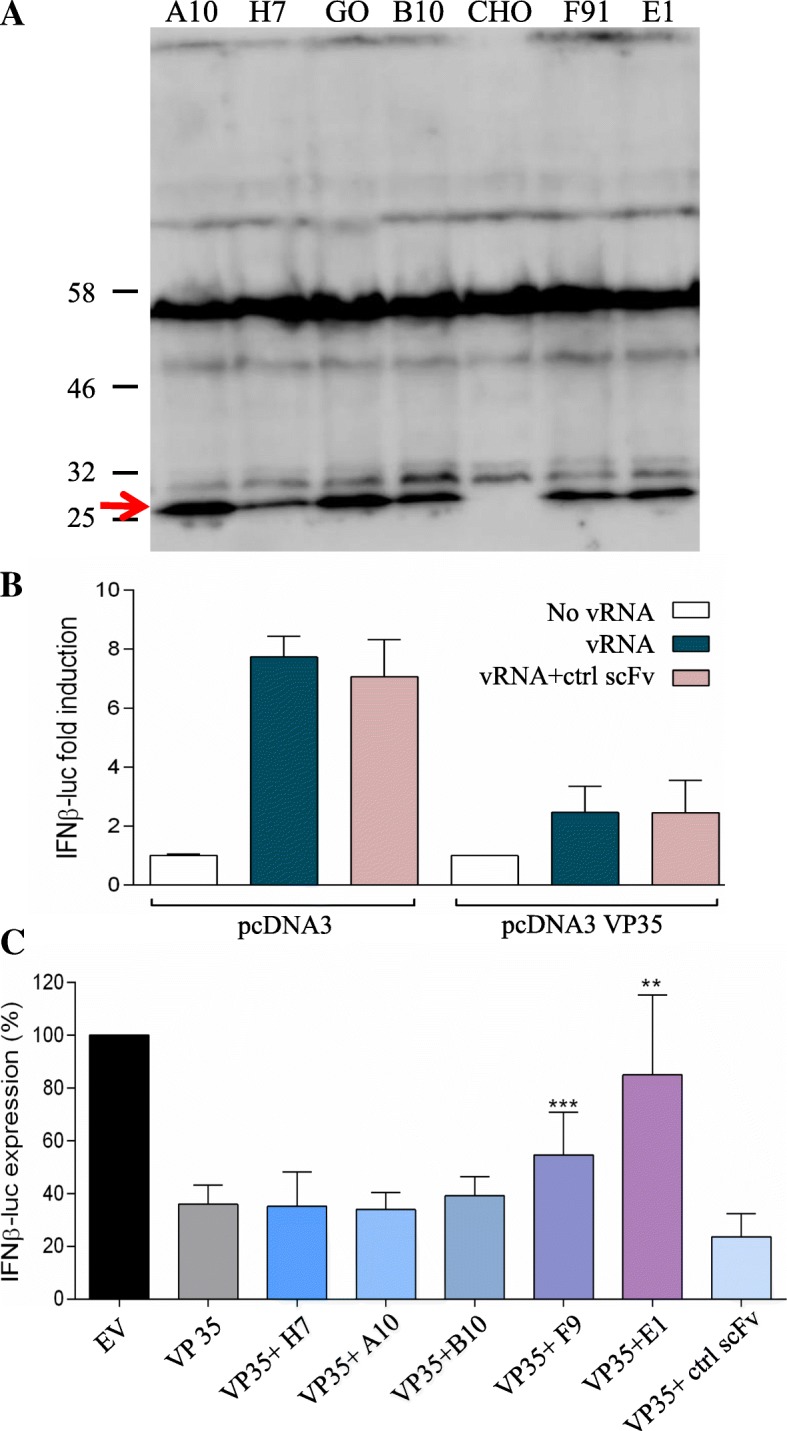
**a** Expression of the monoclonal scFv in eukaryotic cells. WB analysis showing the transient expression of the A10, H7, B10, F9 and E1 scFvs in CHO cells 72 h after transfection with the respective recombinant pTarget plasmids. Specific bands corresponding to scFvs (about 27 kDa) are detected for all clones; other bands of higher molecular mass are present in all the lanes and are a cross-reactivity as evidenced by their presence also in CHO control lane. Molecular mass of the marker is indicated. **b** Controls used in the luciferase reporter gene assay. The histogram shows the effect of transfection procedures on the IFN-β promoter expression. Twenty-four hours after co-transfection with pGL IFN-β luc and pcDNA3 or pcDNA3 EBOV wtVP35 expression vectors, cells were transfected with the ctrl anti-GO scFv pTarget. The next day, cells were additionally transfected with IAV vRNA. The results from four independent experiments performed in triplicate are shown as fold induction of stimulated samples with respect to unstimulated control. **c**. ScFv against EBOV VP35 protein effect in the luciferase reporter gene assay. Twenty-four hours after co-transfection with pGL IFN-β luc and pcDNA3 or pcDNA3 EBOV wtVP35 expression vector, cells were additionally transfected with different scFv p Target vectors and next day additionally transfected with IAV vRNA. The results from three independent experiments are shown as a percentage of the IFNβ promoter induction. Bars indicate the mean ± SD; asterisks indicate a significant difference: **P < 0.01 and ***P < 0.005 (two-tailed unpaired Student’s t-test, *n* = 3)

In order to use the scFvs in the EBOV VP35 luciferase reporter gene inhibition assay, the scFv genes selected were PCR amplified with opportune oligonucleotides and cloned into the pTarget vector for expression in the eukaryotic system. In view of the cytoplasmic VP35 localization, it was not necessary to provide the scFvs with signal sequences for expression in specific cell compartments. Transfection experiments were performed in the CHO cells to evaluate the functionality of the scFv pTarget constructs as described in Methods. The A10, H7, B10, F9 and E1 constructs were all able to express scFvs of the expected molecular mass in eukaryotic cells (Fig. 4, panel a).

### Evaluation of the anti-EBOV VP35 scFvs ability to restore the IFN-β activity by a luciferase reporter gene inhibition assay

To evaluate the capability of the scFvs B10, A10, E1, F9 and H7 to block the VP35 activity, the cell-based miniaturized luciferase reporter gene inhibition assay, previously described [12], was used. The assay measures the capacity of the VP35 expression to inhibit the IFN-β induced by dsRNA treatment, in A549 cells. Before performing the EBOV VP35 luciferase reporter gene inhibition assay using the scFvs, it was crucial to exclude that the expression of an irrelevant scFv could influence the IFN-β induction, either in the absence or in the presence of VP35 expression. To this end, A549 cells, transfected with the pGL IFN-β luc and the pcDNA3-EBOV-VP35 expression plasmid, were co-transfected with the irrelevant anti-GO scFv expression plasmid. Further, to exclude any non-specific effect due to the transfection procedure, the cells were co-transfected in parallel with the pGL IFN-β luc expression vector and an empty pcDNA3 vector.

The luciferase signal emitted in the case of pGL IFN-β luc and anti-GO scFv concurrent expression, was comparable to that obtained with the IFN-β positive controls. Also, in the case of EBOV VP35 and anti-GO scFv simultaneous expression, the measured IFN-β signal was comparable to that obtained with the EBOV VP35 expressed alone (Fig. 4, panel b).

The results confirmed that IFN-β induction was affected by neither the transfection procedure nor the irrelevant scFv either in the presence or in the absence of VP35 expression.

Next, we tested all the scFv pTarget constructs in the dsRNA RIG-I-mediated luciferase reporter gene inhibition assay. Two of the five scFvs tested, F9 and E1, showed a significant ability (*p* = 0.0003 and *p* = 0.0099, respectively) to subvert the inhibition of the IFN-β production generated by dsRNA, after the VP35 expression. By contrast, scFv H7, A10 and B10 showed no ability to counteract the inhibition of IFN-β induction mediated by EBOV VP35 in the cellular assay (Fig. 4, panel c).

### Competitive ELISA using the scFv-expressing phage

**Fig. 5:**
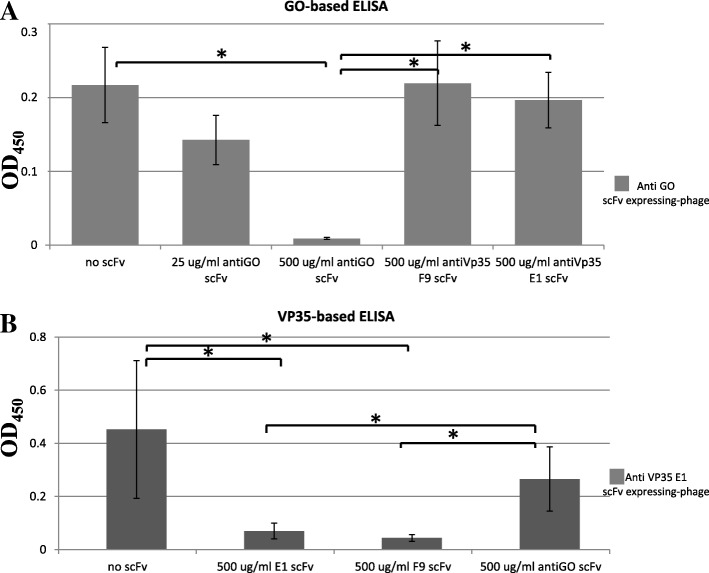
Competitive ELISA for characterization of the scFv binding to EBOV VP35. The binding of the scFv-expressing phages (5 × 10^9^ tu/ml) was measured, both in the absence and in the presence of the competitor soluble non-phage-fused scFvs at the maximum concentration of 500 μg/ml. An anti -M13 PVIII coat protein mAb conjugated to HRP was used for phage detection. On the x-axis the concentrations of scFvs used for the competition assay are reported, on the y-axis, the OD_450_ phage ELISA values are reported. **a** Competitive ELISA using the anti-GO scFv-expressing phages. The binding of anti-GO scFv-expressing phages to plate coated with GO protein is shown: in the absence of competitor soluble non-phage-fused scFvs; in the presence of their own anti-GO soluble non-phage-fused scFvs at the concentration of 500 μg/ml or 25 μg/ml; in the presence of the anti-VP35 F9, and E1 soluble non-phage-fused scFvs, at the concentration of 500 μg/ml. The higher concentration of anti-GO soluble non-phage-fused scFvs shows to compete with itself. The mean values and the SD of an experiment performed in triplicate are reported. Binding in the presence of soluble non-phage-fused scFvs was compared to binding in presence of the control soluble non-phage-fused scFvs or in their absence using Student’s *t*-test. *p < 0.05; **b** Competitive ELISA using anti-EBOV VP35 scFv E1 expressing-phages. The binding of E1 anti-VP35 scFv-expressing phages to plate coated with VP35 protein is shown: in the absence of competitor soluble non-phage-fused scFvs; in the presence of their own E1 soluble non-phage-fused scFvs at the concentration of 500 μg /ml; in the presence of F9 soluble non-phage-fused scFvs at the concentration of 500 μg/ml; in the presence of the control anti-GO soluble non-phage-fused scFvs at the concentration of 500 μg/ml. The mean values and the SD of an experiment performed in triplicate are reported. Binding in the presence of soluble non-phage-fused scFvs was compared to binding in the presence of the control soluble non-phage-fused scFvs or in their absence using Student’s *t*-test. *p < 0.05

To verify whether the two different scFvs E1 and F9, able to subvert the inhibition of the IFN-β production, targeted different epitopes, we performed a competitive ELISA (Fig. 5). This relies on detection of the scFv-expressing phage particles that compete with soluble non-phage-fused scFvs for binding to the antigen immobilized on ELISA plate. It was not possible to detect the soluble non-phage-fused scFvs using the anti-tag Flag ab because the tag is also present on the phage.

To determine the best scFv expressing-phage concentration for competition tests, identified as 5 × 10^9^ tu/ml, we first performed a phage ELISA experiment in which different scFv-expressing phage concentrations were tested on VP35 coated plates (data not shown).

In a competitive assay, ELISA plates coated with VP35 or with the control antigen GO were blocked; they were then incubated with purified scFv-expressing phage both in the absence and in the presence of the competitor soluble non-phage-fused scFv at the maximum concentration of 500 μg/ml. The binding of the scFv-expressing phages was measured. The detection step was via the phage coat protein using an anti-M13 mAb conjugated to HRP. The anti-GO scFv-expressing phage clone was assayed against the anti-GO soluble non-phage-fused scFv as a positive control for competitive measurement (Fig. 5, panel a). It was found that 500 μg/ml of soluble non-phage-fused scFv could inhibit the binding of the scFv-expressing phage in a dose-dependent manner.

Binding in the presence of specific anti-GO soluble non-phage-fused scFv was compared: to binding in the presence of E1 anti-VP35 soluble non-phage-fused scFvs (*p* value = 0.019); to binding in the presence of F9 anti-VP35 soluble non-phage-fused scFvs (*p* value =0.035); to binding in the absence of soluble non-phage-fused scFvs (*p* value = 0.0282) using Student’s *t*-test.

Next, we assayed the binding of E1 scFv-expressing phages both in the absence and in the presence of the competitor soluble non-phage-fused scFv against itself as an intrinsic positive control, against anti-GO soluble non-phage-fused scFv as a negative control and against F9 soluble non-phage-fused scFv for competitive measurement. The inhibiting activity was determined at a single fixed concentration of 500 μg/ml. F9 soluble non-phage-fused scFv shows a clear competitive effect at the concentration used (Fig. 5, Panel b).

Binding in the presence of F9 soluble non-phage-fused scFv was compared to the binding in the presence of anti-GO soluble non-phage-fused scFv (*p* value = 0.0159) and in the absence of soluble non-phage-fused scFvs (*p* value = 0.0182) using Student’s *t*-test.

As a further confirmation, we performed a one-shot experiment by detecting the competition suffered by the F9 scFv-expressing phages when co-incubated with E1 soluble non-phage-fused scFvs and with the control anti-GO soluble non-phage-fused scFv at the concentration of 500 μg/ml. Also in this case we observed that the control anti-GO soluble non-phage-fused scFv has no competitive binding effect. The E1 soluble non-phage-fused scFv competes with the binding of F9 scFv expressing-phages.

Binding in the presence of E1 soluble non-phage-fused scFvs was compared to the binding in the presence of anti-GO soluble non-phage-fused scFv (*p* value = 0.0231) and to binding in the absence of soluble non-phage-fused scFvs (*p* value = 0.0337) using Student’s *t*-test (data not shown).

## Discussion

The urgency to find effective counter measures for the EBOV Disease (EVD) was strengthened by the West Africa outbreaks occurring in 2014–2016, which resulted in 28,646 cases of Ebola with 11,323 deaths [31], pushing the international scientific community to investigate the widest possible range of defense strategies to counteract the virus. However, the current Ebola outbreak, which started in May 2018 in Democratic Republic of the Congo (DRC) and has caused 1016 cases and 634 deaths to date [32], received only minor benefits from the use of new diagnostic assays, vaccines and drugs. The reason is that to control Ebola outbreaks, transversal coordination between health facilities and communities is essential to achieve rapid isolation of the cases of the disease and to stop the transmission chain. Detection of new cases at an early stage by rapid diagnostic tests and ring vaccination strategy with experimental vaccines on volunteers are therefore crucial for controlling EBOV in the current DRC outbreak [33].

Due to the variable onset of antibody response in the Ebola-infected subjects, serology is not used in the acute EVD diagnosis. Conversely, virus and viral proteins accumulate in blood to detectable levels within a few days from disease onset. Molecular tests based on the detection of viral proteins were developed and proved to be effective for diagnosis in acute infection [34]. Currently, in the case of suspected EBOV infection, the World Health Organization (WHO) recommends a list of validated tests for detection of either viral RNA by RT PCR or viral antigens by immunological tests [http://www.who.int/medicines/ebola-treatment/emp_ebola_diagnostics/en/].

Most of the antigen-capture tests used in National Reference Laboratories [35] utilize mAbs generated in mice immunized with the recombinant NP [36], VP40 [37] or GP [38] EBOV proteins. During the recent outbreak, lateral flow immunoassays (LFIs) emerged as powerful tools for rapid antibody-mediated antigen-capture practicable at the point of care [39]. This confirmed the advantages of tests based on antigen-antibody reaction over RT-PCR methodology, which requires significant laboratory infrastructures often lacking in low-income countries. Furthermore, in order to control the transmission-chain of the infection and limit virus spread, it is important to have tests that can be easily automated; as such, antigen capture tests meet this requirement.

EBOV VP35 is a validated drug target for which only a few small molecules have been reported to be active [27, 40, 41], albeit no drug has yet been approved. Here, we present 5 mAbs in scFv format, which are able to react with the Zaire EBOV VP35 protein. This is a key viral protein whose action starts at an early stage of the infection and is based on interference with the host immune response by blocking the IFN-mediated antiviral activity.

The antibodies presented here enrich the list of available anti-VP35 antibodies. They could be used individually or in combination to develop novel reagents for EBOV VP35 detection and therapeutics.

Regarding therapy, pools of neutralizing antibodies have been used in passive immunization of individuals with acute infection [42]; nevertheless, antibodies specific for the VP35 would act with a different mechanism with respect to neutralizing antibodies targeting surface glycoproteins. Recent studies showed that targeting VP35 by either nucleic acid mimics or siRNAs, provides protection against the EBOV infection in murine [43] as well as in non-human primate models [44]. However, the effectiveness of antibodies targeting the VP35 has not yet been demonstrated in vivo.

Here, we show that two out of five scFv antibodies selected against the VP35 significantly hindered the inhibition of the RIG-I signaling cascade mediated by VP35, in a cellular system. We characterized the binding of these two scFv clones to the recombinant VP35 antigen by competitive ELISA, and found that their epitope is at least partly shared. A more detailed analysis of the binding epitopes could further define whether it is a total or partial sharing and which antigenic region of EBOV VP35 is involved in the inhibition of the interferon pathway.

Regarding the other anti-VP35 scFvs selected, we cannot exclude that the observed lack of functionality is due to incorrect scFv folding in the cytoplasmic environment. As antibodies are usually produced in an oxidizing biochemical environment with the help of ER-based chaperones, only a fraction of naïve antibodies can be folded correctly in the reducing cytoplasmic environment which prevents the formation of disulfide bridges.

However, many examples of successful intrabody-mediated proteins knockdown in vitro, obtained using cytosolic intrabodies, have been reported in literature [45].

ScFvs selected in the extracellular environment were previously reported to work intracellularly [12, 20, 22] depending on intrinsic biophysical characteristics such as stability, mainly ascribable to the scaffold. However, several methods have been developed to address the issue of cytosolic intrabodies functioning [45]. Interestingly, anti-VP35 scFvs able to interfere with VP35 activity, were recently isolated from a phage library different from the ETH-2 and were linked to a cell-penetrating peptide for intracytoplasmic delivery [46]. Therefore, although we used a different delivery system, our data strengthen the idea that intracellular antibodies in scFv format can be used to counteract EBOV VP35 activity.

ScFvs against different intracellular EBOV targets could be used either to develop a well-defined cocktail of antibodies with different specificities or also in combination with other drug molecules for therapeutic purposes, provided that an appropriate delivery system is developed.

Furthermore, the VP35 amino acid sequence is highly conserved among the Zaire EBOV isolated in several outbreaks (> 98.2% amino acids identity). Therefore, it can be hypothesized that the scFvs selected retain a broad-spectrum activity.

Nevertheless, the efficacy of these new antibodies should be further evaluated either in Ebolavirus infected cells or in animal models of Ebolavirus infection.

## Conclusion

Five scFv antibodies against an active form of the Zaire EBOV VP35 were isolated and characterized. Their specific reactivity in ELISA and WB suggests the possibility of developing novel reagents for EBOV Vp35 detection during the virus life cycle. The two anti-VP35 scFvs F9 and E1 proved to be able to interfere with the VP35-depending inhibition of IFN activity in a cell system, so suggesting that such antibodies also represent potential therapeutic agents. Further investigations into an EBOV infection system in vitro and in animal models are necessary to validate these reagents.

## Methods

### ETH-2 antibody phage library

The synthetic library of recombinant human antibodies (ETH-2) consists of about 10^8^ scFv polypeptides displayed on the surface of the M13 phage. The library was built by random mutagenesis of the complementarity-determining region 3 (CDR3) of the variable domains of both the heavy (VH) and light (VL) chain of Immunoglobulins, using only three antibody germline gene segments (DP47 for the VH, DPK22, and DPL16 for the VL). In the VH, diversity was created by randomizing four to six positions replacing the pre-existing positions 95 to 98 of the CDR3; in the VL, diversity was obtained by randomizing six positions (91 to 96) of the CDR3 [28].

### Isolation of phage antibodies from ETH-2 library, ELISA and WB

An aliquot of the ETH-2 library, containing 10^12^ cfu phage, was used to isolate specific human antibodies in scFv format against the recombinant VP35 protein (24**)**. Immunotubes (Nunc Maxisorp; Denmark) were coated overnight (ON) at room temperature (RT) with purified recombinant VP35 protein (15 μg/ml in PBS). After panning, phages were eluted with 1 ml of 100 mM triethylamine and the solution was immediately neutralized by adding 0.5 ml of 1 M Tris-HCl pH 7.4. The eluted phages were used to infect an *E. coli* TG1 strain in a log phase, and amplified for the next round of selection, as described in Flego et al. 2005 [30]. Three rounds of panning were performed to recover VP35-specific antibody phages from the ETH-2 library. For the preparation of soluble anti-VP35 scFvs, individual colonies were grown in 96 flat bottomed wells (Nunc) for 2 h at 37 °C in 180 μl of 0.1% glucose 2xYTA medium and induced with 50 μl of 6 mM IPTG/2xYTA medium. The following day, the plates were spun down at 1800 g for 10 min, and the supernatants containing soluble scFvs were recovered and tested for specificity with purified VP35 in ELISA and WB, as described in Flego et al. 2005 [30].

### DNA characterization and sequences

Plasmid DNA from individual bacterial colonies clones was extracted using the Quiaprep spin miniprep Kit and subjected to enzymatic restriction; sequence analysis of the CDR3 regions was then performed with an automated DNA sequencer (BioFab, Roma, Italy) using the fdseq1 (5′-GAA TTT TCT GTA TGA GG-3′) and pelBback (5′-AGC CGC TGG ATT GTT ATT AC-3′) primers.

### Cloning of anti-EBOV VP35 scFvs in pTarget and expression in CHO cells

The scFv gene clones reacting with the recombinant VP35 in ELISA, were PCR amplified using the following primers:

ETH2 NCO 1 as a forward primer: 5′ GCGC acc atg gcc gag gtg cag ctg 3′.

NHE I STOP HIS as a reverse primer: 5′ GCGC gct agc cta atg atg atg atg atg atg tgc ggc cgc gcc tag gac 3′ containing the 6xHis tag sequence.

For transient expression in eukaryotic cells, the amplimers were cloned in pTarget (PROMEGA) under the strong viral promoter (Cytomegalovirus immediate-early enhancer). The clones obtained were sequenced to check for mutations possibly introduced by PCR, and used to transfect CHO cells using JetPei DNA transfection reagent, according to the manufacturer’s instructions.

Transiently transfected cells were lysed after 48 h with SDS-loading buffer (50 mM Tris-HCl pH 6.8, 3% SDS, 5% 2-mercaptoethanol, 50% glycerol), loaded onto 12% SDS-PAGE, and then transferred to a nitrocellulose membrane using standard procedures. The membrane was blocked in 2% MPBS ON at RT. Blotted proteins were incubated for 2 h in 2% MPBS with 1 μg/ml of Tetra·His Antibody (Qiagen), which was the only anti-His mAb able to recognize the tag at the scFv COOH terminus, in our experimental conditions. After an additional incubation for 1 h at RT in the presence of goat anti-mouse antibody HRP-conjugate (5 μg/ml, Dako), the reaction was developed and visualized with a chemiluminescence detection kit (Pierce; IL, USA).

### Luciferase reporter gene assay

#### IFN-β induction luciferase reporter gene assays

A549 cells (5 × 10^4^ per well) were transfected in 48-well plates with T-Pro P-Fect Transfection Reagent (T-Pro Biotechnology) with the construct pGL IFN-β luc, kindly provided by Prof. Stephan Ludwig (Institute of Molecular Virology, Münster, Germany). Twenty-four hours after transfection, cells were additionally transfected using IAV PR8 vRNA and incubated for a further 6 h at 37 °C with 5% CO_2_. Cells were harvested with lysis buffer (50 mM Na-MES pH 7.8, 50 mM Tris-HCl pH 7.8, 1 mM dithiothreitol, 0.2% Triton X-100). The crude cell lysates were cleared by centrifugation and 50 μL of cleared lysates were added to 50 μl of luciferase assay buffer (125 mM Na-MES pH 7.8, 125 mM Tris-HCl pH 7.8, 25 mM magnesium acetate, 2.5 mg/ml ATP) in a white 96-well plate. Immediately after addition of 50 μl of 1 mM D-luciferin into each well, the luminescence was measured in Victor3 luminometer (Perkin Elmer). The relative light units (RLU) were normalized as the fold activity of the unstimulated control. Each assay was carried out in triplicate.

#### EBOV VP35 luciferase reporter gene inhibition assay

The above described luciferase reporter gene assay was also performed for evaluating the IFN-β induction inhibition mediated by EBOV VP35. Twenty-four hours after co-transfection with pGL IFN-β luc and pcDNA3 or pcDNA3 EBOV wtVP35 expression vectors, cells were transfected with the ctrl anti-GO scFv pTarget as an irrelevant scFv and with the different scFv p Target vectors The next day, cells were additionally transfected with IAV vRNA. Inhibition of luciferase expression was indicated either as the fold activity of the unstimulated control or as a percentage of the induced control. Each assay was carried out in triplicate.

### Competitive ELISA using scFv-expressing phages

For ELISA competition assay, we used soluble non-phage-fused scFvs produced and purified as in Gellini et al. [47]. ScFv-expressing phages were produced from a monoclonal bacterial culture grown to OD_600 =_0.4–0.5 and infected with M13K07 helper phage in a ratio of around 20: 1 phage/bacteria. One hundred ml of supernatant containing scFv-expressing phages were 50x concentrated by precipitation with PEG 6000 and resuspended in PBS. Phage titer was determined by plating of bacteria infected with phages at scalar dilutions. Coating was performed with VP35 or GO antigen as described in the ELISA section. The following day, the plate was blocked with 2% MPBS for 2 h at RT and washed with TPBS. Twenty-five μl of soluble non-phage-fused scFvs at two-fold the desired final concentration were pre-incubated in 1% MPBS with the antigen for 15 min prior to the addition of 25 μl of the scFv-expressing phage mix at two times the desired final tu/ml in 1% MPBS, and incubated for 1 h at RT. The plate was washed with TPBS and incubated with anti-M13 PVIII coat protein mAb conjugated to HRP (Amhersham) diluted 1: 1000, for 1 h RT. A washing step was conducted followed by the addition of peroxidase substrate as described above.

## Data Availability

The datasets used and/or analyzed during the current study are available from the corresponding author on reasonable request.

## Abbreviations

EBOV: Ebola filovirus
ECL: Enhanced chemiluminescence
ELISA: *enzyme-linked immunosorbent assay*
GO: Glucose oxidase
HRP: Horseradish peroxidase
IFN: Interferon
IPTG: Isopropyl β-dithiogalactopyranoside
kDa: Kilodaltons
mAb: Monoclonal antibody
MPBS: non-fat dry milk in PBS
OD: Optical density
ON: Overnight
PBS: Phosphate buffered saline
RT: Room temperature
scFv: Single-chain fragment variable
SD: Standard deviation
SDS-PAGE: Sodium dodecyl sulfate-polyacrylamide gel electrophoresis
TPBS: 0.05% Tween 20 in PBS
VP35: Viral protein 35
